# Analytical Analysis of Recirculating Flow in Single-Screw Extruders

**DOI:** 10.3390/polym17212959

**Published:** 2025-11-06

**Authors:** Chris Rauwendaal

**Affiliations:** Rauwendaal Extrusion Engineering, Inc., 25126 Rodeo Flat, Auburn, CA 95602, USA; chris@rauwendaal.com

**Keywords:** recirculating, extruder, single-screw extruder, Newtonian fluid

## Abstract

Current analytical theories of recirculating flow in single-screw extruders consider only cross-channel flow in channels of infinite width with only one exception. Proper analysis of recirculating flow requires inclusion of normal velocities and the effect of finite channel width. More broadly, this paper presents an analytical description of lid-driven cavity flow—one of the most frequently studied flows in fluid dynamics. Expressions for velocities and flow rates for Newtonian fluids are obtained that satisfy the balance equations. These expressions have been compared to results of numerical analyses with good agreement. Flow rates and velocities are displayed with 3D surface plots and contour plots. These plots provide better insight into the flow behavior than 2D graphs. We have analyzed flow in slit channels with width much greater than the height (W>>H) and flow in a square channel (W=H). The vortex center (stagnation point) in a slit channel is located at normal coordinate ψ=2/3. The vortex center in a square channel is located at ψ=0.76. These analytical results allow for the development of better analytical models for melt temperature distribution, mixing, and devolatilization in single-screw extruders.

## 1. Introduction

In this paper, we examine the cross-channel flow of molten polymers in single-screw extruders. Current analytical extrusion theory describes the tangential velocities at the flow centerline for rectangular channels of infinite width. Current theory does not describe flow in channels with finite width.

We will present an analytical description of recirculating flow that includes both cross-channel and normal velocity components at all positions in the channel. We will present expressions for flow in channels with infinite width (slit channels) as well as channels with finite width. More broadly, this paper presents an analytical description of lid-driven cavity flow—one of the most frequently studied flows in fluid dynamics. Most studies of lid-driven flow use numerical methods. A comprehensive review of these studies with 335 references was published by Kuhlman and Romano [1].

**Figure 1:**
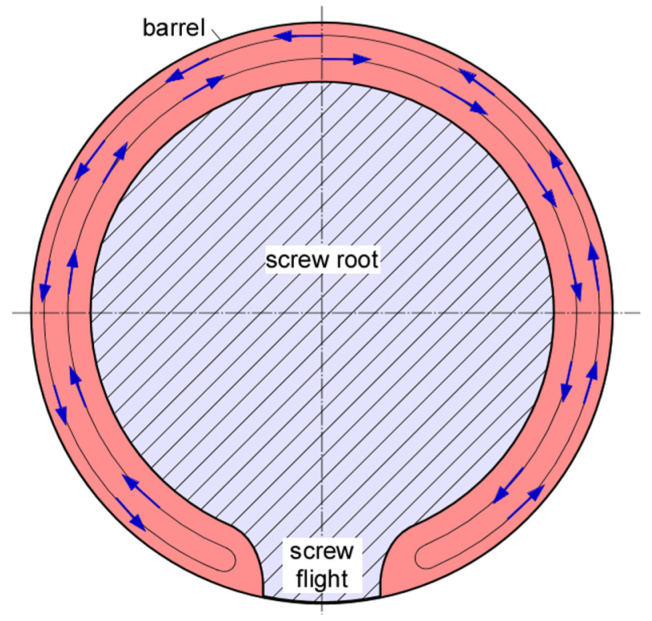
Screw and barrel cross section perpendicular to screw axis. The blue arrows show the movement along one streamline.

We will consider a stationary screw with the barrel rotating around the screw in counterclockwise direction, see Figure 1.

The circumferential velocity $v_{b}$ of the barrel is πDN where π is 3.14159…, D is the barrel diameter, and N is the rotational speed. The flow is assumed to be in steady state; inertia, centrifugal, and gravitational forces are assumed negligible and the fluid viscosity and density constant. This type of flow is referred to as creeping flow. The Reynolds number associated with this flow is zero, Re = 0.

In this analysis, this geometry is simplified using the flat plate approximation [2,3]. The annular channel is be approximated with a rectangular channel with width W and height H. The analysis is based on the fluid adhering to the screw and barrel surfaces without wall slip. It is assumed that the viscosity is temperature-independent.

## 2. Current Theory of Recirculating Flow in Single-Screw Extruders

The starting point of analysis of fluid flow is the Navier–Stokes Equation (1). The Navier–Stokes equation for incompressible Newtonian fluid in steady state can be expressed as

(1) $$\rho \left(\vec{v}\cdot \nabla \right)\vec{v}=-\nabla P+\mu \nabla^{2}\vec{v}$$

We will examine creeping flow where inertia forces are negligible. For this flow, the Reynolds number is zero (Re=0). This means that the left term of the Navier–Stokes equation can be neglected. The continuity equation is

(2) $$\nabla \cdot \vec{v}=0$$

The Navier–Stokes equation for flow in the axial direction (z-direction) relates the axial pressure gradient to the velocity gradients. It can be written as

(3) $$\frac{\partial P}{\partial z}=\mu \nabla^{2}v_{z}=\mu (\frac{\partial {v_{z}}^{2}}{\partial x^{2}}+\frac{\partial {v_{z}}^{2}}{\partial y^{2}})$$

The pressure gradients that govern the recirculating flow relate to the velocity gradients as well. The tangential pressure gradient (x-direction) can be written as

(4) $$\frac{\partial P}{\partial x}=\mu \nabla^{2}v_{x}=\mu (\frac{\partial {v_{x}}^{2}}{\partial x^{2}}+\frac{\partial {v_{x}}^{2}}{\partial y^{2}})$$

The normal pressure gradient (y-direction) can be expressed as

(5) $$\frac{\partial P}{\partial y}=\mu \nabla^{2}v_{y}=\mu (\frac{\partial {v_{y}}^{2}}{\partial x^{2}}+\frac{\partial {v_{y}}^{2}}{\partial y^{2}})$$

These elliptic partial differential equations for the pressure gradients are often referred to as Poisson’s equation. The first analysis of transverse flow in single-screw extruders was published by Mohr et al. [4] for a channel with infinite width (W>>H). In this case, the $\partial v_{x}/\partial x$ term in Equation (4) is set to zero ($\partial v_{x}/\partial x=0$). The tangential velocity for zero leakage flow can be written as

(6) $$v_{x}(y)=-2v_{b}y/H+3v_{b}y^{2}/H^{2}$$

The pressure gradient for zero leakage flow can be expressed as

(7) $$\frac{\partial P}{\partial x}=\frac{6\mu v_{b}}{H^{2}}$$

**Figure 2:**
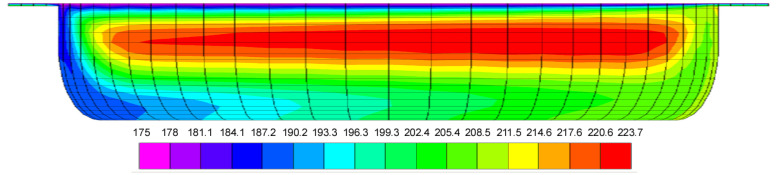
Color contour plot of melt temperature distribution in a single-screw extruder [13].

Kaufman et al., published analyses of recirculating flow [5,6]. The analysis by Mohr et al. [4] has been incorporated in many textbooks, for instance, Bernhardt [7], Schenkel [8,9], McKelvey [10], and Tadmor and Klein [11]. Many papers have been published on 3D flow in single-screw extruders using computational fluid dynamics, CFD. Liao published a numerical analysis of recirculating flow [12]. Rauwendaal [13] published an analysis of non-isothermal flow of a power law fluid in single-screw extruders including leakage flow over the flight. He found that the recirculating flow tends to produce a region of high temperature in the center region of the channel. An example of this high-temperature region is shown in the color contour plot of the melt temperatures in the channel cross-section in Figure 2. The left side of the channel is the pushing flight flank; the right side of the channel is the trailing flight flank. The top is the barrel surface, and the bottom is the root of the screw.

**Figure 3:**
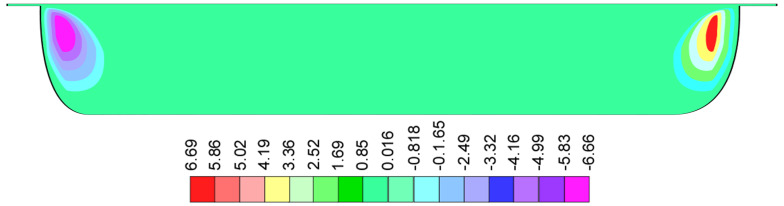
Color contour plot of normal velocities [13] in a cross-section of the screw channel of a single screw extruder.

It is interesting to note that the stock temperatures in the flight gap are lower than those in the high-temperature region even though the shear rates in the flight gap are much higher than those in the channel. The normal velocities [13] are shown in Figure 3 as a color contour plot of the cross-section of the screw channel.

Normal velocities only occur close to flight flanks. The width of the normal velocity region is about 0.6 H. The velocities are negative (downward) at the pushing flight flank and positive (upward) at the trailing flight flank.

Examples of CFD analyses are Sanjabi [14], Vachagina [15], Sikora [16], and Syrjälä [17]. Lid-driven cavity flow was studied by Burggraf et al. [18], Rubin et al. [19], Gia et al. [20], and many others as reviewed by Kuhlman and Romano [1]. Analyses of 2D isothermal recirculating cross-channel flow have been published by Carley et al. [21], Squires [22], and Fenner [23].

The continuity equation is written as

(8) $$\frac{\partial v_{x}}{\partial x}+\frac{\partial v_{y}}{\partial y}=0\;\;$$

The recirculating flow is completely determined by a stream function Ψ(x,y) [3]. The tangential velocity is found by taking the first derivative of the stream function with respect to normal coordinate y.

(9) $$v_{x}=-\frac{\partial \Psi }{\partial y}$$

Similarly, the normal velocity is found by taking the first derivative of the stream function with respect to tangential coordinate x.

(10) $$v_{y}=-\frac{\partial \Psi }{\partial x}$$

By inserting the expressions for $v_{x}$ and $v_{y}$ into the expressions for the pressure gradients, we can express the tangential pressure gradient in terms of the stream function:

(11) $$\frac{\partial P}{\partial x}=-\mu \frac{\partial }{\partial y}\nabla^{2}\Psi$$

The normal pressure gradient becomes

(12) $$\frac{\partial P}{\partial y}=-\mu \frac{\partial }{\partial x}\nabla^{2}\Psi$$

The stream function can now be expressed as

(13) $$\nabla^{2}{(\nabla }^{2}\Psi )=0$$

This is a bi-harmonic differential equation. The boundary conditions require the velocities normal to the screw and barrel surface to be zero. The stream function can be expressed as the sum of an irrotational function $\Psi_{i}$ and a rotational function $\Psi_{r}$ so that it satisfies the LaPlace equation. The LaPlace equation can be written as follows:

(14) $$\nabla^{2}\Psi_{i}=0$$

Kaufman [5] developed an analytical model for recirculating flow in single-screw extruders. Kaufman writes the rotational stream function as a third-order polynomial in x and y. He omitted the velocity gradient $\partial v_{x}/\partial x$ and $\partial v_{y}/\partial y$ in his model to avoid singularities in the top corners of the channel. The stream function will satisfy Equation (11). The rotational stream function can be written as

(15) $$\Psi_{r}=a+b_{1}x+b_{2}y+\frac{c_{1}x^{2}}{\alpha }+{c_{2}xy+c}_{3}y^{2}+\frac{d_{1}x^{3}}{\alpha^{2}}+\frac{d_{2}x^{2}y}{\alpha }+d_{3}xy^{2}+d_{4}y^{3}$$

In this expression, the height of the channel H equals one (H=1) and the width of the channel W equals the aspect ratio α=W/H. Kaufman analyzed the case where the rotational stream function contribution is a sum of a function of x only and a function of y only. In this case, $c_{2}=d_{2}=d_{3}=0$. The rotational stream function now reduces to

(16) $$\Psi_{r}=a+b_{1}x+b_{2}y+\frac{c_{1}x^{2}}{\alpha }+c_{3}y^{2}+\frac{d_{1}x^{3}}{\alpha^{2}}+d_{4}y^{3}$$

The LaPlace equation can be solved by the method of separation of variables. This yields an expression for the irrotational stream function involving products of trigonometric and hyperbolic functions. One combination is

(17) $$\left[Asin\left(k_{1}x\right)+Bcos\left(k_{1}x\right)\right]\left[Csinh\left(k_{1}y\right)+Dcosh\left(k_{1}y\right)\right]$$

Another combination is

(18) $$\left[Esin\left(k_{2}y\right)+Fcos\left(k_{2}y\right)\right]\left[Gsinh\left(k_{2}x\right)+Hcosh\left(k_{2}x\right)\right]$$

The values of $k_{1},\;k_{2}$; A, B, C, D, E, F, G; and H are determined using the boundary conditions. The stream function can be written as

(19) $$\Psi =A+B+a-\left(c_{1}+d_{1}\right)x-\left(c_{3}+d_{4}\right)y+\frac{c_{1}x^{2}}{\alpha }+c_{3}y^{2}+\frac{d_{1}x^{3}}{\alpha^{2}}+d_{4}y$$

Factor *A* is an infinite series:

(20) $$A=\sum_{n=1}^{n=\infty }\frac{4}{\pi^{3}n^{3}}\left[c_{3}-{\left(-1\right)}^{n}\left(c_{3}+3d_{4}\right)\right]\frac{\cosh\left[n\pi \left(x-\alpha /2\right)\right]}{cosh(0.5n\pi /\alpha )}\sin\left(n\pi y\right)$$

Factor A is a summation of $F_{A}\;(n,x,y)$, where n goes from n=1 to n→∞. That may seem like a daunting task. Fortunately, only a few terms are required to achieve accurate results. We will use a summation of n=1 to n=5 to calculate the stream function. Factor *B* is also an infinite series:

(21) $$B=\sum_{n=1}^{n=\infty }\frac{4w}{\pi^{3}n^{3}}\left[c_{1}-{\left(-1\right)}^{n}\left(c_{1}+3d_{1}\right)\right]\frac{\cosh\left[\frac{n\pi \left(y-1/2\right)}{\alpha }\right]}{cosh(0.5n\pi /\alpha )}\sin\left(\frac{n\pi x}{\alpha }\right)$$

Kaufman [5] sets $c_{1}=d_{1}=0$. This means that factor B=0.

**Figure 4:**
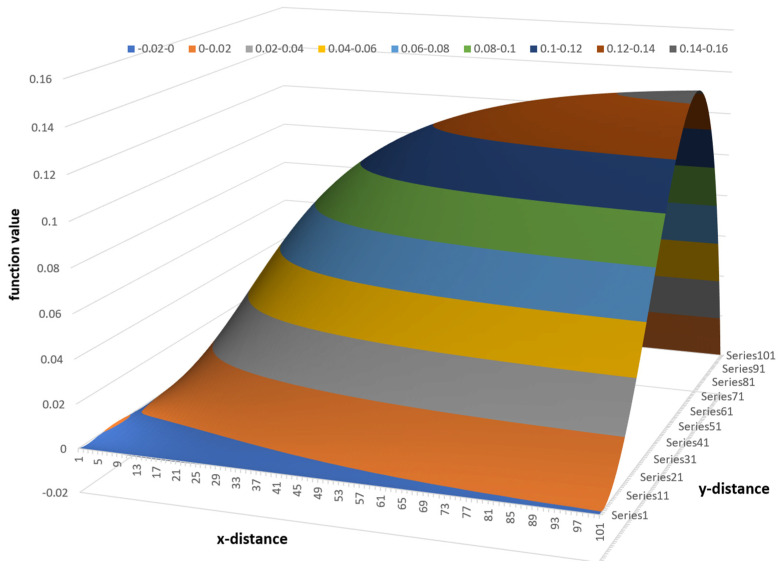
A 3D surface plot of the stream function for a channel with an aspect ratio of 10:1.

Figure 4 shows the 3D surface plot of the stream function for a channel with an aspect ratio of 10:1.

**Figure 5:**
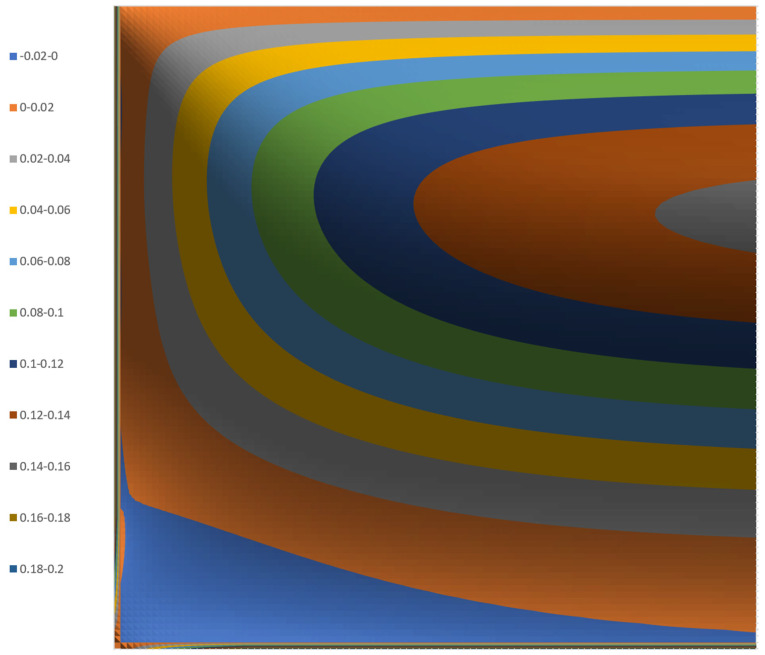
A 3D contour plot of the stream function for a channel with an aspect ratio of 10:1.

The surface plot was obtained by summation of $F_{A}\;(n,x,y)\;$ from n=1 to n=5. The streamlines do not change significantly beyond n=3. Therefore, the computations can be simplified. The streamlines can be shown more clearly in the contour plot as shown in Figure 5.

These figures show negative values of the stream function in the lower left corner of the channel. These indicate that the calculations of the stream function are not entirely correct. The figures also show non-zero values at the side wall (flight flank) and bottom wall (screw root). These values indicate that the predicted values are not consistent with zero velocities at the walls.

The pressure gradient is found by substituting the stream function in Equations (10) and (11). The tangential pressure gradient becomes

(22) $$\frac{\partial P}{\partial x}=-6d_{4}\mu$$

The normal pressure gradient becomes

(23) $$\frac{\partial P}{\partial y}=6d_{1}\mu /\alpha$$

The circulation along a closed curve in the x, y plane of the rectangular channel is obtained by using the Stokes theorem and Equations (22) and (23).

(24) $$\Gamma =\oint_{xy\;loop}\vec{v}[]dl=\iint \left(\nabla \times v\right)dxdy=\int_{0}^{w}\int_{0}^{1}\nabla^{2}\Psi dxdy=\alpha \left(2c_{3}+3d_{4}\right)+2c_{1}+3d_{1}$$

For a slit channel with W >> H, constants $c_{1}$ and $d_{1}$ can be set to zero ($c_{1}=d_{1}=0$). The tangential x-velocity can now be expressed as

(25) $$v_{x}(x,y)=A_{x}+c_{3}(1-2y)+d_{4}(1-3y^{2})$$

where

(26) $${\;\;\;\;\;\;\;\;\;\;\;A}_{x}=-\sum_{n=1}^{n=\infty }\frac{4}{\pi^{2}n^{2}}\left[c_{3}-{\left(-1\right)}^{n}\left(c_{3}+3d_{4}\right)\right]\frac{\cosh\left[n\pi \left(x-\alpha /2\right)\right]}{\cosh\left(0.5n\pi \alpha \right)}\cos\left(n\pi y\right)$$

The y-velocity can be written as

(27) $$\;v_{y}\left(x,y\right)=\sum_{n=1}^{n=\infty }\frac{4}{\pi^{2}n^{2}}\left[c_{3}-{\left(-1\right)}^{n}\left(c_{3}+3d_{4}\right)\right]\frac{\sinh\left[n\pi \left(x-\alpha /2\right)\right]}{\cosh\left(0.5n\pi \alpha \right)}\sin\left(n\pi y\right)$$

Constants $c_{3}$ and $d_{4}$ can be determined by the boundary condition $v_{x}(x=0.5w,y=0)=0$ and $v_{x}\left(x=0.5w,y=1\right)=-v_{bx}$. We neglect the exponentially small terms of the order $e^{-\pi w}$. This leads to the following expressions:

(28) $$c_{3}=-v_{bx}(1-\frac{8}{\pi^{2}}e^{-0.5\pi \alpha })$$

and

(29) $$d_{4}=v_{bx}$$

The pressure gradient in Equation (22) becomes $\partial P/\partial x\approx -6\mu v_{bx}$. This is the result of the analytical solution for channels of infinite width. It should be remembered that length is expressed in units of height H. The circulation is $\Gamma \approx \alpha v_{bx}$, where terms of the order of $e^{-0.5\pi \alpha }$ are neglected. This is approximately equal to the circulation on the boundary loop assuming non-slip boundary conditions.

## 3. Dividing the Channel in 1D and 2D Sections

**Figure 6:**
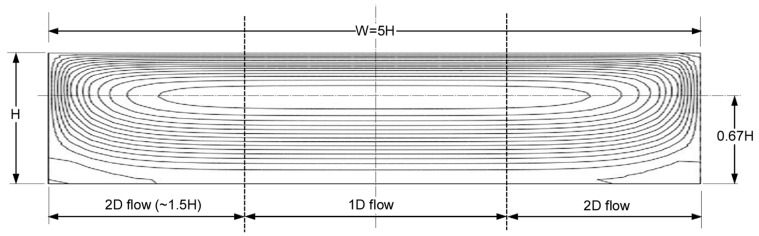
Streamlines for a channel with a 5:1 aspect ratio with proper height and width dimensions.

Figure 6 shows the streamlines determined by Kaufman [5] for a channel with an aspect ratio of 5:1.

The flow in the center region of the channel for all practical purposes is 1D flow—the tangential velocities are non-zero and the normal velocities are zero. In this region, the streamlines are parallel to the barrel surface. The edge regions have both tangential and normal velocities—the streamlines have distinct curvature. The curved streamlines indicate that the edge regions have 2D flow.

The 2D flow at the edges occurs over a width of approximately 1.5 H from the side walls. The center region is dominated by 1D flow. With W/H=5, the width of the 1D flow region is about 2 H. It can be reasonably assumed that the width of the 2D flow region is independent of the W/H ratio for channels with ratios above 3:1. The aspect ratio (width-to-height) is represented by Greek letter alpha (α=W/H).

This means that the width of the normal flow region is approximately 1.5 H. For slit flow with W/H>3:1, the width of the normal flow region is less than half the width of the channel. The normal velocities $\varphi_{y}$ are expressed as a third-order polynomial of the dimensionless x-coordinate ξ. At the left wall ξ=0 and at the center of the channel ξ=0.5, see also Figure 6.

(30) $$\varphi_{y}=b_{3}\xi^{3}+b_{2}\xi^{2}+b_{1}\xi +b_{0}$$

Coefficient $b_{0}$ is zero because $\varphi_{y}$ equals zero at the side wall, $\varphi_{y}\left(0\right)=0$. The flow rate is obtained by integrating the velocity function. This results in the following expression for the normal flow rate:

(31) $${\dot{V}}_{y}={0.25b}_{3}\xi^{4}+b_{2}\xi^{3}/3+{0.5b}_{1}\xi^{2}$$

The normal flow rate is normalized relative to the rightward flow rate ${\dot{V}}_{\to }$:

(32) $$\Phi_{y}=\frac{{\dot{V}}_{y}}{{\dot{V}}_{\to }}={1.6875b}_{3}\xi^{4}+{2.25b}_{2}\xi^{3}+{3.375b}_{1}\xi^{2}$$

The shear rate (velocity gradient) is obtained by taking the first derivative of the velocity:

(33) $${\dot{\gamma }}_{y}={3b}_{3}\xi^{2}+2b_{2}\xi +b_{1}$$

The normal velocities must satisfy the following boundary conditions:

- The velocity must be zero at ξ=1.5/α;
- The shear rate must be zero at ξ=1.5/α;
- The flow rate at ξ=1.5/α must equal the rightward flow rate ${\dot{V}}_{y}\left(1.5/\alpha \right)=4/27$.

With these boundary conditions, the coefficients of the polynomial of the normal velocity function can be determined.

(34a) $$b_{1}=-0.7901\alpha$$

(34b) $$b_{2}=1.0535\alpha^{2}$$

(34c) $$b_{3}=-0.3512\alpha^{3}$$

**Figure 7:**
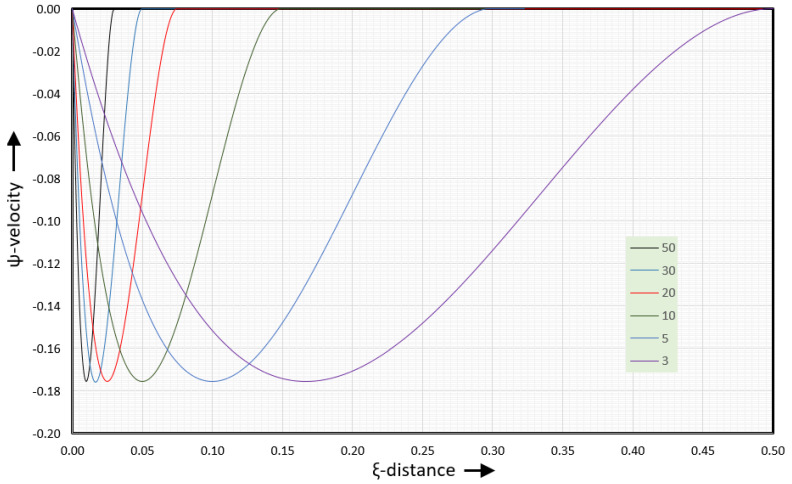
y-velocities for several values of the aspect ratio.

Figure 7 shows the ψ-velocities for several values of the aspect ratio.

The minimum value of the normal velocities is −0.176 when the barrel velocity is set at $v_{b}=-1$. The shape of the velocity curve is that of a wave. For an aspect ratio of 50:1, the normal velocities are only non-zero in the first and last 6% of the channel width. For an aspect ratio of 10:1, the normal velocities are non-zero in the first and last 15% of the channel width. For an aspect ratio of 3:1, the normal velocities are non-zero over the full width of the channel, except right in the middle of the channel. If the α=3 curve is compressed horizontally by a factor of 10, the result will be the α=30 curve.

The ψ-velocities for aspect ratio values less than 3 are the same as those for an aspect ratio of 3. However, when the aspect ratio is less than 3, the tangential velocities and the position of the vortex center (stagnation point) change with the aspect ratio. In this case, the assumption that the channel width is much larger than the channel height (W >> H) is no longer valid. In this case, the channel must be treated as a channel with finite width.

## 4. Velocities Along Vortex Centerline

**Figure 8:**
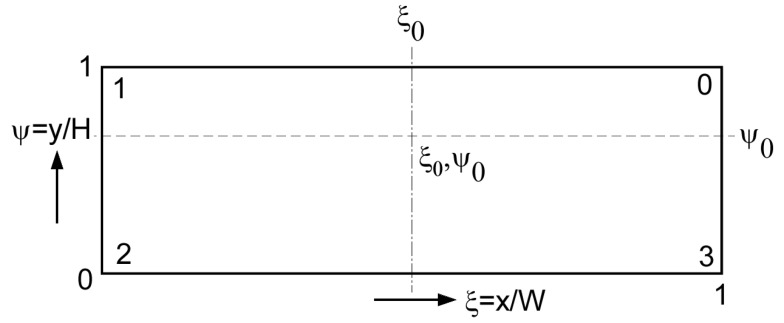
Dimensionless coordinate system.

It is convenient to work with dimensionless coordinates, see Figure 8.

The dimensionless x-coordinate is ξ=x/W, where W is the width of the channel. The dimensionless y-coordinate is ψ=y/H, where H is the height of the channel. The velocity is made dimensionless by dividing the velocity by the barrel velocity, $\varphi_{x}=v_{x}/v_{b}$ and $\varphi_{y}=v_{y}/v_{b}.$ The flow rate is made dimensionless by dividing the flow rate by the rightward flow rate (${\dot{V}}_{\to }$), The dimensionless tangential flow rate is $\Phi_{x}={\dot{V}}_{x}/{\dot{V}}_{\to }$. The dimensionless normal flow rate is $\Phi_{y}={\dot{V}}_{y}/{\dot{V}}_{\to }$. The dimensionless coordinate system is shown in Figure 8.

The tangential velocity in the center of the channel is zero at $\psi_{0}$. For a rectangular channel where the width W of the channel is much greater than the height H of the channel (W>>H), the value is $\psi_{0}=2/3.$ The normal velocities are zero at $\xi_{0}=0.5$. The center of the recirculating flow is located at $\xi_{0},\;\psi_{0}$. The value of $\psi_{0}$ changes as fluid elements move away from the centerline.

We will first consider a rectangular channel where the width W of the channel is much greater than the height H of the channel (W>>H). We will use the term slit channel for such a channel. There is an analytical solution for the tangential velocities in recirculating flow in a slit channel. The tangential velocities along the vortex centerline can be written as follows [1,4]:

(35) $$\varphi_{x}=2\psi -3\psi^{2}$$

**Figure 9:**
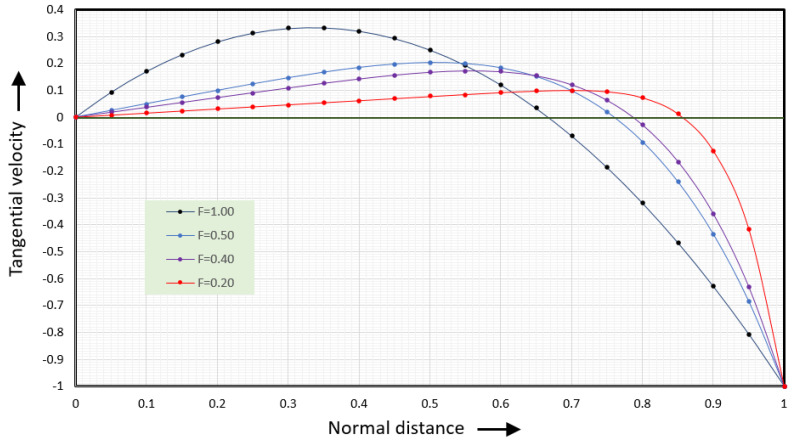
Tangential velocity versus normal distance for several value of the dimensionless flow rate F.

This is the dimensionless form of Equation (6) for the tangential velocity $v_{x}(y)$. Figure 9 shows the tangential velocity versus normal distance.

The black line (F = 1.00) shows the velocities along the vortex centerline where the dimensionless normal flow rate equals 1. The blue line (F = 0.50) shows the velocities for a dimensionless normal flow rate of 0.5. The purple line (F = 0.40) shows the velocities for a dimensionless normal flow rate of 0.4 and the red line (F = 0.20) for a dimensionless normal flow rate of 0.2. The tangential velocities at values of the dimensionless flow rate less than one (F < 1) will be discussed in the next section.

When the dimensionless normal flow rate equals 1 the tangential velocity is zero at the root of the screw, ψ=0, and at ψ=2/3;  the velocity at the barrel surface is $\varphi_{x}(1)=-1$. The maximum rightward velocity is 1/3, $\varphi_{xmax}(\psi =1/3)=1/3$. This maximum occurs at one-third the distance from the bottom of the screw channel.

In slit flow, the velocities in the center region of the channel are dominated by the cross-channel velocities. The normal velocities become significant as fluid elements approach the side walls, but this region occupies only a small fraction of the channel. The tangential flow rate in slit flow along the vortex centerline is obtained by integration of the velocities; this leads to

(36) $${\dot{V}}_{x}=\left(\psi^{2}-\psi^{3}\right)$$

The rightward flow rate ${\dot{V}}_{\to }$ is ${\dot{V}}_{x}\left(2/3\right)=4/27$. The dimensionless flow rate is the tangential flow rate divided by the rightward flow rate.

(37) $$\Phi_{x}={\dot{V}}_{x}/{\dot{V}}_{\to }=a_{\Phi }(\psi^{2}-\psi^{3})$$

**Figure 10:**
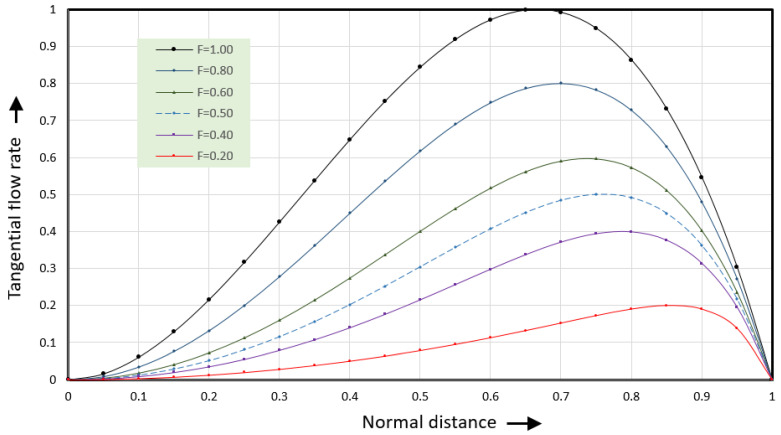
Tangential flow rate versus normal distance for six values of the dimensionless normal flow rate F.

Coefficient $a_{\Phi }$ equals $a_{\Phi }=27/4=6.75$. The tangential shear rate is obtained by taking the first derivative of the velocities. Figure 10 shows the tangential flow rate versus normal distance.

The black line (F = 1.00) shows the velocities along the vortex centerline where the dimensionless normal flow rate equals 1. The dark blue line (F = 0.80) shows the velocities for a dimensionless normal flow rate of 0.8. The green line (F = 0.60) shows the velocities for a dimensionless normal flow rate of 0.6. The light blue dashed line (F = 0.50) shows the velocities for a dimensionless normal flow rate of 0.5. The purple line (F = 0.40) shows the flow rate for a dimensionless normal flow rate of 0.4 and the red line (F = 0.20) for a dimensionless normal flow rate of 0.2.

The tangential flow rates at values of the dimensionless flow rate less than one (F < 1) will be discussed in the next section. The shear rates can be expressed as

(38) $${\dot{\gamma }}_{x}\left(\psi \right)=2-6\psi$$

**Figure 11:**
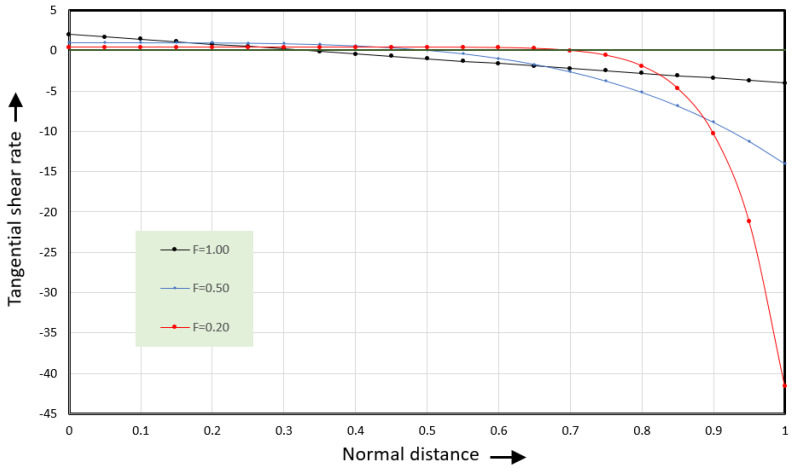
Tangential shear rate versus dimensionless normal coordinate ψ.

The shear rate is zero at ψ=1/3. This is also the location where the rightward velocity reaches a maximum. Figure 11 shows the shear rates versus normal distance.

The shear rates along the vortex centerline (F = 1.00) are positive in the lower 1/3 of the channel and negative in the top 2/3 of the channel. The shear rate at the root of the screw (ψ = 0) equals ${\dot{\gamma }}_{x}\left(0\right)=2$ s^−1^. The shear rate at the barrel (ψ = 1) equals $\;{\dot{\gamma }}_{x}\left(1\right)=-4$ s^−1^. The shear rate at the barrel is twice the shear rate at the root of the screw. As a result, the shear stress at the barrel is twice the shear stress at the root of the screw.

The shear rates at lower values of the dimensionless normal flow rate (F = 0.50 and F = 0.20) will be discussed in the next section.

The expressions for the normal flow rate and velocities at the flow centerline $\psi_{0}$ must meet the following requirements:

- The flow rate is zero at ξ=0  and ξ=1;
- The flow rate is 1 at ξ=0.5;
- The velocity must be zero at ξ=0  and ξ=0.5;
- The downward flow rate equals the rightward flow rate and the upward flow rate;
- The velocity gradient at ξ=0.5 must be zero.

The normal pressure gradient varies with tangential distance. It is maximum at the flight flanks (ξ=0 and ξ=1) and reduces to zero in the middle of the channel (ξ=0.5). If the normal pressure gradient is a linear (first-order) function of tangential distance, the shear rate will be a second-order function, the velocity a third-order function, and the flow rate a fourth-order function of tangential distance. The normal flow rate at the flow centerline can be expressed as

(39) $$\Phi_{y}\left(\xi ,\psi_{0v}\right)={24\xi }^{2}-64\xi^{3}+{48\xi }^{4}$$

**Figure 12:**
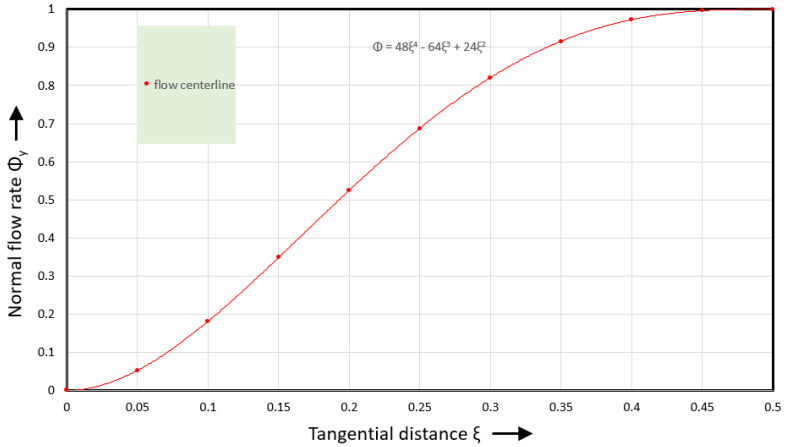
Normal flow rate versus tangential distance.

Figure 12 shows the normal flow rate versus tangential distance.

The red flow rate curve starts at zero slope, the slope increases to a maximum value at ξ~0.2, and then reduces to zero at ξ=0.5. The normal velocity is obtained by taking the first derivative of the normal flow rate. This leads to the following expression:

(40) $$\varphi_{y}\left(\xi ,\psi_{0v}\right)=(48\xi -192\xi^{2}+192\xi^{3})b_{\varphi }$$

where $b_{\varphi }=4/27=0.148148$.

**Figure 13:**
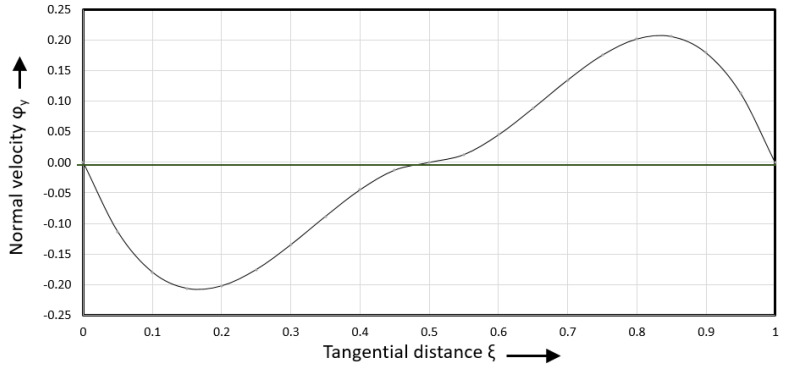
Normal velocity versus tangential distance.

Figure 13 shows the normal velocity versus tangential distance.

The normal velocities reach a minimum at ξ=1/6 and a maximum at ξ=5/6. The normal velocity is zero at ξ=0, ξ=0.5, and ξ=1. The normal shear rate is obtained by taking the first derivative of the normal velocity. This leads to the following expression:

(41) $${\dot{\gamma }}_{y}\left(\xi ,\psi_{0v}\right)=-7.1111+56.8888\xi -{85.3333\xi }^{2}$$

**Figure 14:**
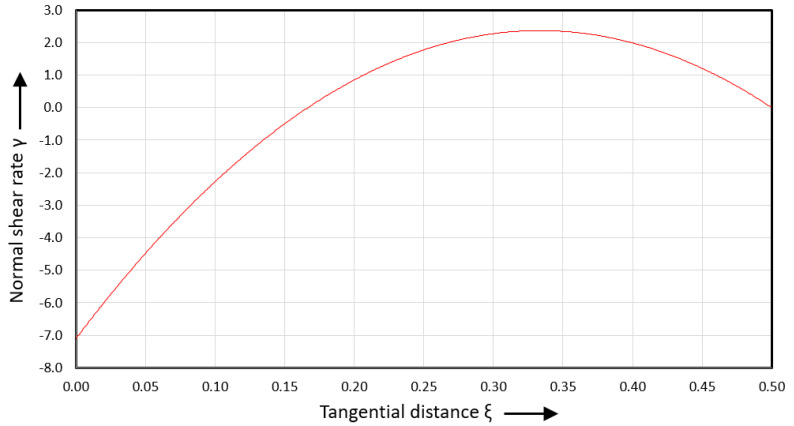
Normal shear rate versus tangential distance.

Figure 14 shows the normal shear rate versus tangential distance.

The normal shear rate is negative from ξ=0 to ξ=1/6 and positive from ξ=1/6 to ξ=0.5. The normal shear rate is zero at ξ=1/6 and ξ=0.5. The normal velocity reaches its minimum value at ξ=1/6 and its maximum value at ξ=5/6.

## 5. Velocities Away from Vortex Centerline for Slit Flow

The velocities reduce as fluid elements move closer to the flight flanks. Figure 10 shows tangential flow rates versus normal distance at several values of the dimensionless normal flow rate. The tangential flow rate curves must have the following properties:

- The flow must be zero at ψ=0 and ψ=1;
- The maximum value must equal the maximum value of the normal flow rate;
- The location of the maximum value must increase with reducing normal flow rate;
- The location of the maximum value most approach 1 as normal flow rate approaches 0;
- The slope of the flow rate curve must be −1 at ψ=1 because the velocity $\varphi_{x}\left(1\right)=-1$.

A generalized expression for the tangential flow rate can be obtained by modifying the expression valid for the vortex centerline, Equation (37). The power of the second ψ-term is modified to $3/\Phi_{y}$. The coefficient $a_{\Phi }$ now becomes a function of the normal flow rate $\Phi_{y}$.

(42) $$\Phi_{x}(\xi ,\psi )=a_{\Phi }\Phi_{y}(\psi^{2}-\psi^{3/\Phi_{y}})$$

Evaluation of this expression requires an expression of the normal flow rate as a function of tangential distance ξ—see Equation (39). Coefficient $a_{\Phi }$ can be expressed as

(43) $$a_{\Phi }=a_{3}{\Phi_{y}}^{3}+a_{2}{\Phi_{y}}^{2}+a_{1}\Phi_{y}+a_{0}$$

where $a_{3}=8.291$, $a_{2}=-7.9618$, $a_{1}=5.7492$, and $a_{0}=0.6673$.

At this point, we can determine the flow rate as a function of tangential distance ξ and normal distance ψ. Figure 9 shows the tangential flow rate at six values of the dimensionless normal flow rate.

**Figure 15:**
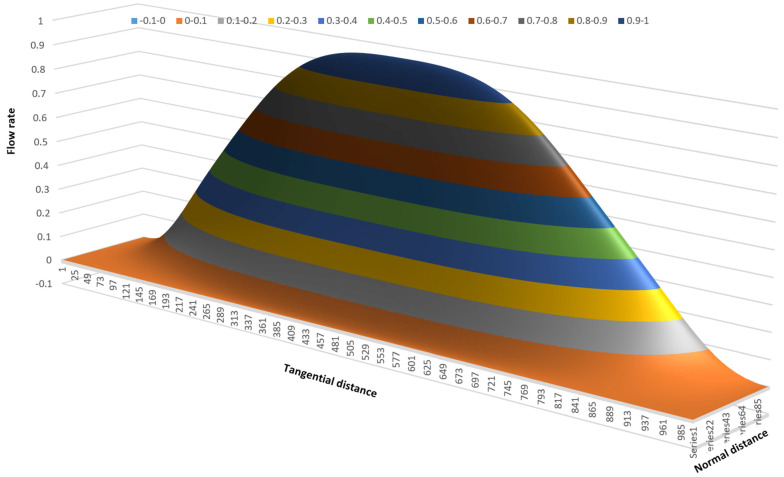
Three-dimensional surface plot of flow rate versus x- and y-distance.

The variation in flow rate relative to the position in the channel can be visualized effectively using a 3D surface plot. One advantage of a 3D surface plot is that it can be viewed from multiple angles. Figure 15 shows a 3D surface plot of the flow rate versus x- and y-distance viewed from the bottom of the channel. The surface plot was constructed using a 100 × 1000 matrix with 100 steps in the vertical direction and 1000 steps in the horizontal direction.

Figure 15 shows the flow rate in a channel with an aspect ratio of 10:1; the width of the channel is 10 times the height of the channel. The different colors indicate regions with different flow rates. In this case, ten different flow rate regions are shown. The lowest flow rates range from 0 to 0.1; the highest flow rates range from 0.9 to 1.0. The lowest flow rates occupy the largest part of the channel.

The flow rate surface is shaped like a mountain gently sloping up from the screw surface with a steep slope at the barrel surface. The maximum flow rate is located at ξ=0.5 and ψ=2/3.

**Figure 16:**
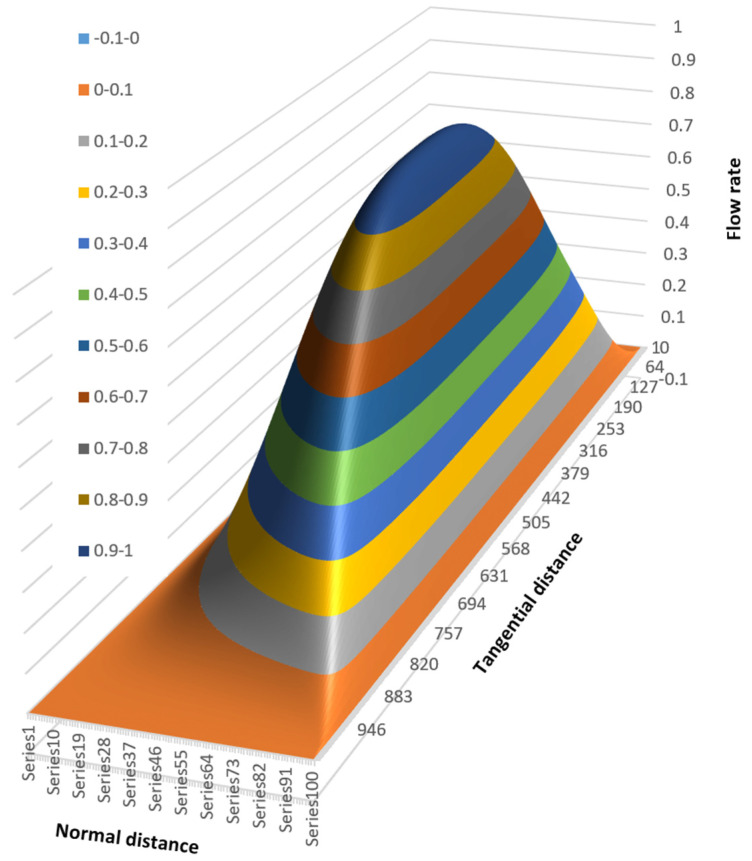
Surface plot viewed from the top right corner of the channel.

Figure 16 shows the surface plot viewed from the top right corner of the channel.

**Figure 17:**
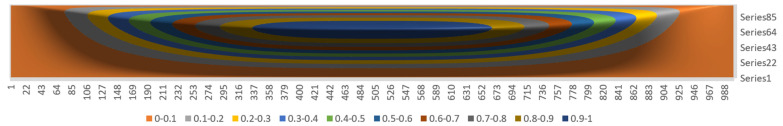
Contour plot of flow rate versus x- and y-distance.

This view clearly shows the steep slope of the flow rate surface plot at the barrel surface. The lines of equal flow rate can be shown clearly in a contour plot, as shown in Figure 17.

The lines of equal flow rate correspond to the streamlines of the flow. The streamlines in the top one-third of the channel are spaced closely together as compared to those in the bottom two-thirds of the channel. The streamlines close to the barrel surface are almost horizontal, while those closer to the screw surface have distinct curvature.

The streamlines have an elliptical shape with the top portion of the ellipse compressed relative to the bottom portion. The width of the ellipse increases with reducing flow rate. The location of the farthest left values of the ellipse moves closer to the top left corner as the flow rate approaches zero. The location of the farthest right values of the ellipse moves closer to the top right corner as the flow rate approaches zero.

The contour plot clearly shows the large region of slow flow rate in the bottom left and right corner of the channel. The low velocities in the bottom corners of the channel are problematic because they result in long residence times. These, in turn, lead to thermal degradation of the polymer.

## 6. Velocities Away from Vortex Centerline for Square Channel

The expressions for slit flow do not apply to channels with finite width. Most studies of lid-driven cavity flow have focused on square channels [8,9]. We will study the flow in a square channel to illustrate the difference in flow characteristics compared to a channel of infinite width. In a channel with finite width, the shear stresses acting on the side walls (flight flanks) will reduce the shear stresses acting on the root of the screw. As a result, the shear rates at the screw surface diminish when the aspect ratio (W/H) of the channel reduces. At the same time, the flow center or stagnation point of the tangential flow moves closer to the barrel surface.

**Figure 18:**
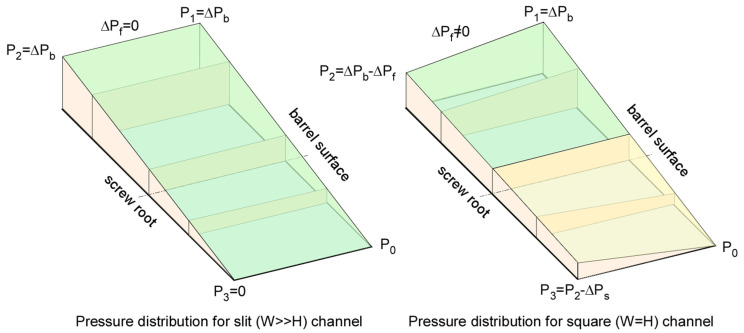
Pressure distribution in slit channel (**left**) and square channel (**right**).

Figure 18 shows qualitatively the pressure distribution for a slit channel (left) and a square channel (right). In slit flow, the pressure drop at the flight flanks is negligible relative to the pressure increase along the barrel. As a result, the increase in pressure along the barrel $∆P_{b}$ equals the pressure drop along the root of the screw $∆P_{s}$, see Figure 18 left.

In a square channel, the pressure drop at the flight flanks is not negligible. In this case, the increase in pressure along the barrel $∆P_{b}$ is greater than the pressure drop along the root of the screw $∆P_{s}$, see Figure 18 right.

**Figure 19:**
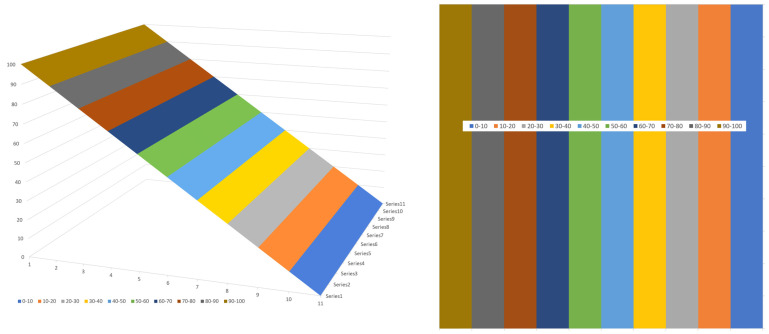
Three-dimensional surface plot (**left**) and contour plot (**right**) of pressures in the slit channel.

Figure 19 shows a 3D surface plot for the pressures in a slit channel on the left and with a contour plot on the right. The pressure at the top of the trailing flight flank is set at zero. The increase in pressure along the barrel is set at 100 and the pressure drop along the flight flanks is set at zero.

In a slit channel, the normal pressure gradients are zero throughout the channel, as indicated by the straight vertical lines in the contour plot. The normal pressure gradients are zero when the pressure drop along the flight flanks is negligible relative to the pressure drop along the barrel.

**Figure 20:**
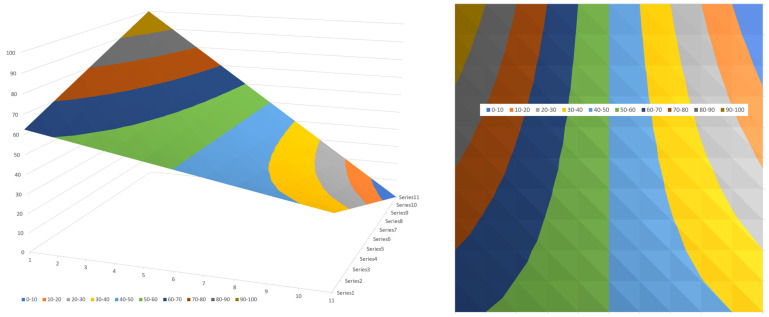
Three-dimensional surface plot (**left**) and contour plot (**right**) of pressures in the square channel.

Figure 20 shows a 3D surface plot for a square channel. The pressure at the top of the trailing flight flank is set at zero. The increase in pressure along the barrel is set at 100 and the pressure drop along the flight flanks is set at 37.5.

The pressure distribution in the square channel is substantially different from that in the slit channel. The normal pressure gradients are non-zero throughout the channel except for the middle of the channel. The lines of equal pressure (isobars) are straight in the slit channel but curved in the square channel.

In a square channel, the pressure drop at the flight flanks is not negligible relative to the pressure increase along the barrel. As a result, the increase in pressure along the barrel, $∆P_{b}$, is greater than the drop in pressure along the root of the screw $∆P_{s}$. The normal pressure gradient is negative in the left side of the channel and positive in the right side of the channel. The negative normal pressure gradient creates downward flow in the left side of the channel. The positive normal pressure gradient creates upward flow in the right side of the channel.

It should be noted that the normal pressure gradient in the square channel is zero at the center of the channel. As a result, there is no normal flow in the center of the channel. The figure showing the slit channel pressure field does not show the pressures close to the side walls. When the channel width is much greater than the channel height, W >> H, the effect of the side walls is negligible. In this case, the flow in the center region of the channel is dominated by tangential flow—this is 1D flow. In a square channel, the side walls have a significant effect on the flow with tangential and normal flow equally important throughout the channel. As a result, in a square channel there is no region with 1D flow.

When the height of the channel increases, the rightward flow rate reduces. With it, the upward, leftward, and downward flow rates reduce as well. This will move the location of the vortex center upward. Numerical studies [8,9] have shown that the vortex center for a square channel is located at ψ=0.76. This location corresponds to the ξ-value where the normal flow rate $\Phi_{y}(\xi )$ in a slit channel is half of the maximum flow rate $\Phi_{y}(\xi =1)$. This leads to the following expression for the tangential velocity along the flow centerline for a square channel:

(44) $$\varphi_{x}(\xi_{0v},\psi )=0.5\psi -1.5\psi^{5}$$

The tangential velocity becomes zero at $\psi_{0v}=0.759835685$. That means that the flow center is located at approximately three-quarters the height of the channel. The average rightward velocity in the square channel is

(45) $${\bar{\varphi }}_{x\to }(\xi_{0v},\psi )=0.12664$$

Figure 9 shows this tangential velocity versus normal distance. The tangential velocity in the square channel is the light blue line (F = 0.50). The maximum rightward velocity in the square channel is about 0.2; the actual maximum velocity is 0.203253. The maximum rightward velocity in the slit channel is 1/3=0.3333 and the average rightward velocity is 2/9=0.2222. The tangential flow rate is found by integrating the tangential velocity. It can be written as

(46) $$\Phi_{x}(\xi ,\psi )=0.25a_{\Phi }(\psi^{2}-\psi^{6})$$

where $a_{\Phi }$ is 10.392312 to make the maximum value of $\Phi_{x}$ equal to one

The rightward flow rate for the square channel is

(47) $${\dot{V}}_{\to }=0.096225$$

The rightward flow rate for the slit channel is 0.148 (4/27). That means that the rightward flow rate in the square channel is about two-thirds the flow rate in the slit channel. The barrel surface $A_{b}$ in the square channel is one-third the screw surface $A_{s}$. In the slit channel, the barrel surface equals the screw surface. We can postulate a relationship between the rightward flow rate and the ratio of barrel surface to screw surface:

(48) $${\dot{V}}_{\to }\left(W,H\right)=2{\dot{V}}_{\to }\left(W\gg H\right)A_{b}/A_{s}$$

This expression would predict a rightward flow rate for the square channel of ${\dot{V}}_{\to }=0.098765$. This is within 3% of the value calculated using Equation (36) and shown in Figure 10. This expression needs to be tested for channels with several values of the W/H ratio.

Figure 10 shows the tangential flow rate for several values of the flow rate $\Phi_{y}$. The blue curve with a dashed line (F = 0.50) becomes the flow rate curve at the flow centerline for the square channel. The tangential shear rate along the flow centerline is found by taking the first derivative of the tangential velocity. This leads to

(49) $${\dot{\gamma }}_{x}(\xi_{0v},\psi )=0.5-7.5\psi^{4}$$

The tangential shear rate becomes zero at $\psi_{0s}=0.508133$. This is the normal location where the tangential rightward velocity reaches a maximum value.

Figure 11 shows the shear rates versus normal distance for slit flow for three values of the flow rate $\Phi_{y}$. The blue curve (F = 0.5) becomes the shear rate curve at the flow centerline for the square channel. General expressions for the flow rate and velocity can be obtained using the same procedure that was used for slit flow. The tangential flow rate can be expressed as

(50) $$\Phi_{x}(\xi ,\psi )=a_{\Phi_{y}}({\Phi_{y}\psi }^{2}-{\Phi_{y}\psi }^{6/\Phi_{y}})$$

The factor $a_{\Phi }$ is a function of the normal flow rate. It can be expressed as

(51) $$a_{\Phi }=0.58{\Phi_{y}}^{3}-0.56{\Phi_{y}}^{2}+1.57\Phi_{y}+1.00$$

**Figure 21:**
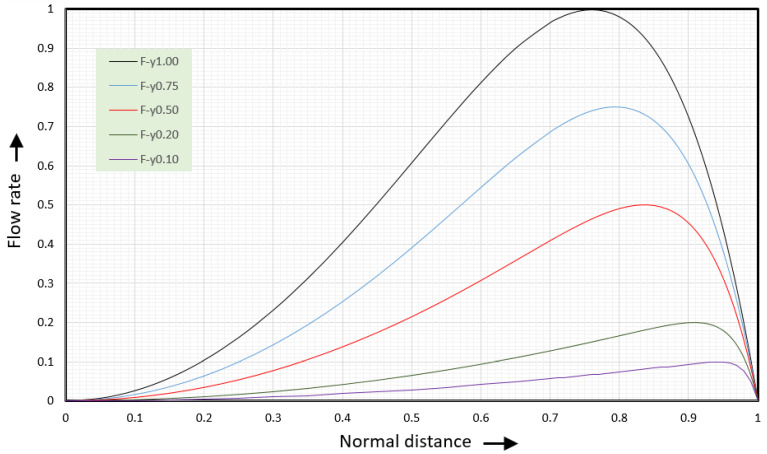
Flow rate versus normal distance for five values of the normal flow rate F.

Figure 21 shows the tangential flow rate versus normal distance for five values of the normal flow rate $\Phi_{y}$. For $\Phi_{y}=1$, the maximum flow rate equals 1 at ψ =0.76. For $\Phi_{y}=0.75$, the maximum flow rate occurs at ψ =0.79. For $\Phi_{y}=0.5$, the maximum flow rate occurs at ψ =0.84. For $\Phi_{y}=0.2$, the maximum flow rate occurs at ψ =0.91. For $\Phi_{y}=0.1$, the maximum flow rate occurs at ψ =0.95.

The general expression for the tangential velocity can be written as

(52) $$\varphi_{x}\left(\xi ,\psi \right)=a_{\varphi }[0.5\Phi_{y}\psi -1.5\psi^{(6/\Phi_{y}-1)}]$$

Factor $a_{\varphi }$ is a function of the normal flow rate. It can be expressed as

(53) $$a_{\varphi }=0.0577{\Phi_{y}}^{3}+0.046{\Phi_{y}}^{2}+0.2303\Phi_{y}+0.666$$

**Figure 22:**
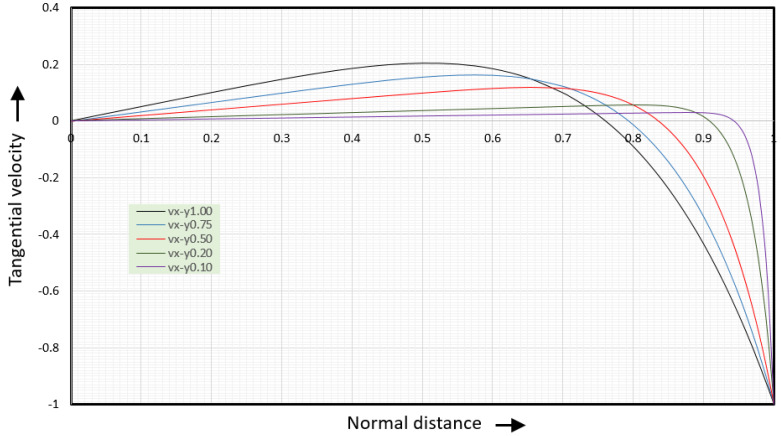
Tangential velocity versus normal distance.

The tangential velocity becomes zero at ψ=0, ${and\;\psi }_{0v}(\Phi )={\left(\Phi /3\right)}^{1/(6/\Phi -1)}$. Figure 22 shows the tangential velocity versus normal distance.

The tangential shear rate is obtained by taking the first derivative of the velocity:

(54) $${\dot{\gamma }}_{x}(\xi ,\psi )=0.5\Phi_{y}-1.5(6/\Phi -1)\psi^{(6/\Phi -2)}$$

The tangential shear rate becomes zero at

(55) $$\psi_{0s}\left(\Phi_{y}\right)={\left(\frac{{\Phi_{y}}^{2}}{18-3\Phi_{y}}\right)}^{1/(6/\Phi_{y}-2)}$$

This is the location where the tangential rightward velocity reaches a maximum.

**Figure 23:**
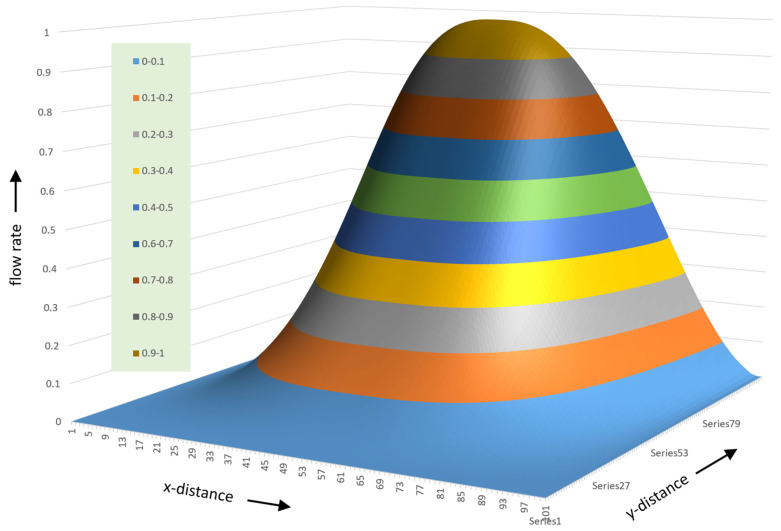
Three-dimensional surface plot of flow rate for square channel viewed from lower right.

The flow rate as a function of ξ and ψ can now be calculated. Figure 23 shows a 3D surface plot of the flow rate viewed from the lower right corner of the channel.

**Figure 24:**
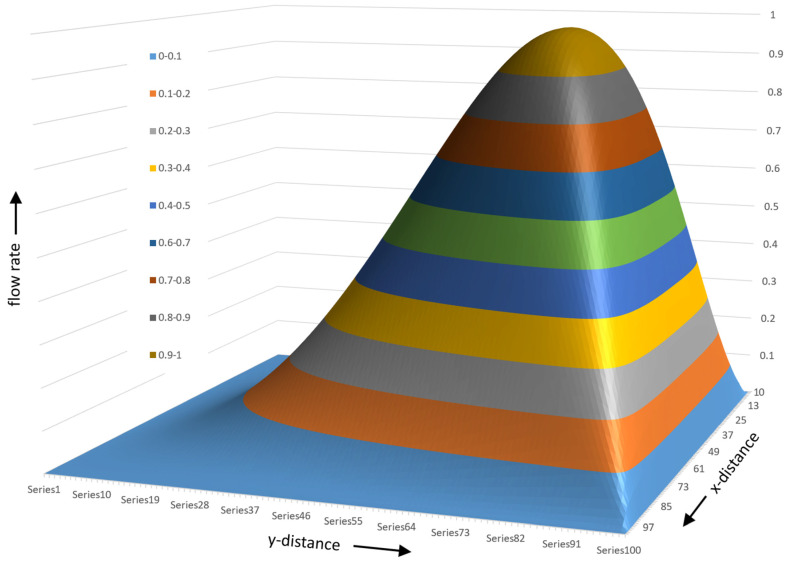
A 3D surface plot seen from the upper right corner of the channel.

Figure 24 shows the surface plot seen from the upper right corner of the channel.

**Figure 25:**
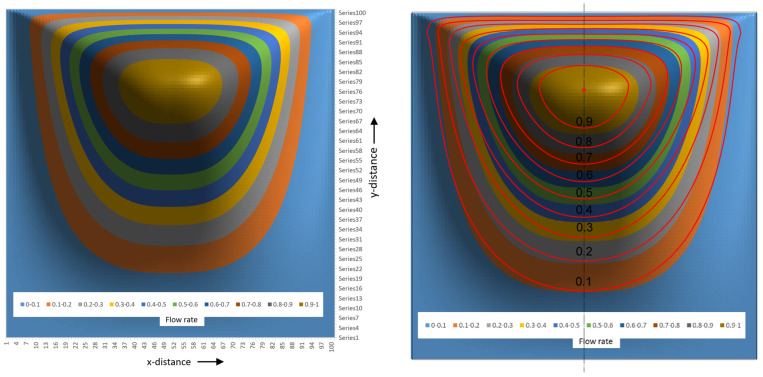
Contour plot of flow rate for the square channel; Liao streamlines added in right image.

The flow rate surface plot for a square channel looks like a mountain with a flat bottom at the screw surface with a gently increasing slope. The barrel side has a steeply sloped surface due to the fact that the barrel velocity is −1. The peak of the surface occurs at ξ=0.5 and ψ=0.76. The surface plot is generated using a 100 × 100 matrix with one hundred steps along the height of the channel and one hundred along the width of the channel. Figure 25 shows the flow rate contour plot for the square channel.

The contour lines represent the flow streamlines in the channel. These streamlines can be conveniently compared to results from numerical studies of lid-driven flow in square cavities. The right side of Figure 25 shows the contour plot with streamlines added determined from a numerical study by Liao [12]. The agreement between analytical and numerical results is close enough to make the analytical results useful for practical applications. The essential features of the recirculating flow are captured by the analytical model.

**Figure 26:**
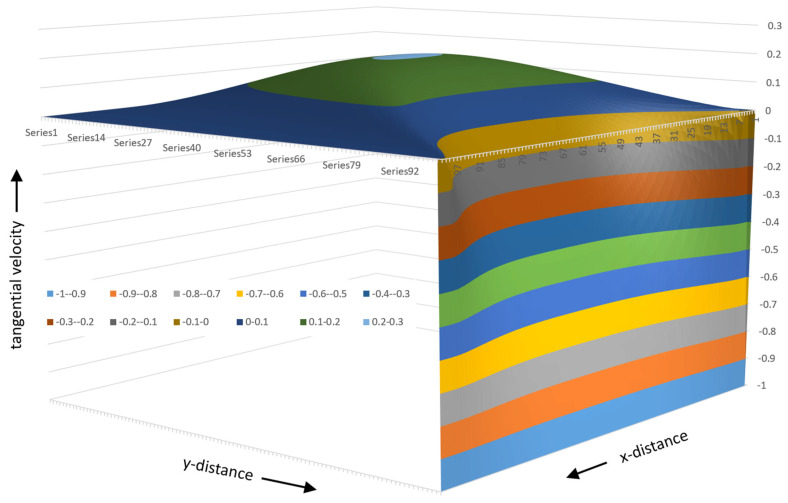
A 3D surface plot of the tangential velocity.

**Figure 27:**
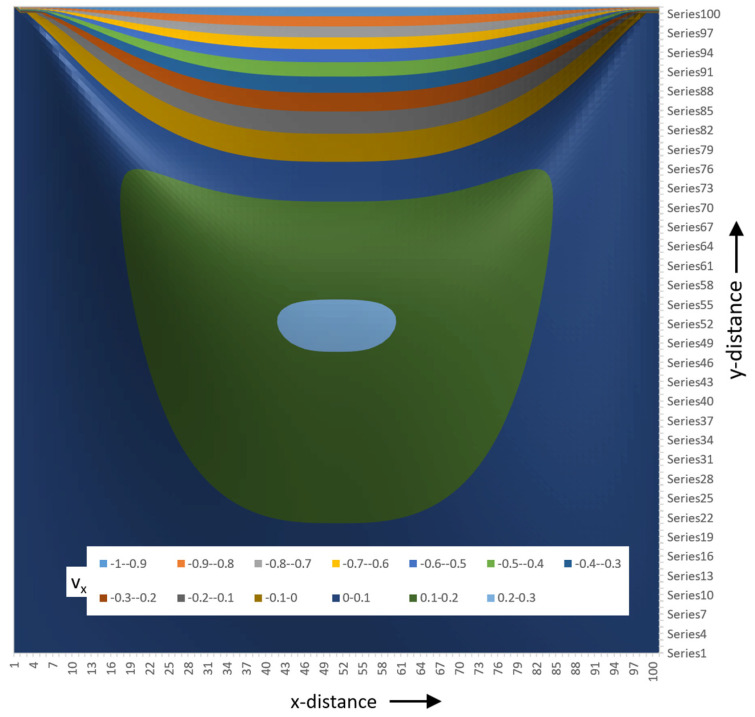
A contour plot of the tangential velocities.

Figure 26 shows the 3D surface plot of the tangential velocity. The tangential velocities are positive in the lower portion (~75%) of the channel and negative in the upper portion (~25%) of the channel. Close to the barrel surface, the velocities reduce rapidly to a value of -1 at the barrel surface. The velocities are between 0 and 0.1 in more than 50% of the channel. That indicates that the fluid velocities are low in most of the channel. This can be seen more easily in the contour plot shown in Figure 27.

Low velocities lead to long residence times. Long residence times lead to polymer degradation. This is an inherent problem in single-screw extruders. The square channel is not optimal with respect to polymer degradation. To reduce degradation, the screw channel should be designed with large radii at the lower corners of the channel.

Figure 27 shows the contour plot of the tangential velocity.

The contour plot of the tangential velocity clearly shows that fluid velocities are low in more than 50% of the channel. Negative velocities occur in the top quarter of the channel. The tangential velocities reduce quickly close to the barrel surface. This can be seen by the fact that color bands of different velocity become increasingly thin close to the barrel.

## 7. Conclusions

In this paper, we developed closed-form, analytical expressions for cross-channel and normal velocities in the recirculating flow of a Newtonian fluid with zero leakage flow. The agreement between analytical and numerical results for the streamlines is acceptable. The agreement is not perfect, but close enough to be useful for most practical applications.

Flow rates and velocities have been displayed with 3D surface plots and contour plots. These plots provide better insight into the flow behavior than 2D graphs and allow for detailed examination of the flow characteristics. We analyzed flow in a slit channel where the width is much greater than the height (W>>H). We also analyzed flow in a square channel (W=H).

The vortex center (stagnation point) in a slit channel is located at normal coordinate ψ=2/3. The vortex center in a square channel is located at ψ=0.76. These analytical results allow for the development of better analytical models for melt temperature distribution, mixing, and devolatilization in single screw extruders. Development of these models is currently underway.

The flow in horizontal slit channels (W>>H) is a combination of 1D flow region in the center and 2D flow region at the edges. The analytical results presented in this paper compare well with results from numerical studies. In channels with finite width (W<3H), 2D flow occurs throughout the volume of the channel—these is no significant region of 1D flow.

The flow in vertical slit channel (W<<H) is a combination of 2D flow in the top layers of the channel and stagnation (0D flow) in the bottom of the channel. The flow in a vertical slit channel is not covered in this study as it has no practical application in extruders used for polymer processing. Stagnation in the bottom of the channel will create problems with polymer degradation. As a result, channels where with the width is much smaller than the height (W<<H), are not practical in screw extruders used for polymer processing.

## Data Availability

The original contributions presented in this study are included in the article. Further inquiries can be directed to the corresponding author.
